# Isolation of *CHS* Gene from *Brunfelsia acuminata* Flowers and Its Regulation in Anthocyanin Biosysthesis

**DOI:** 10.3390/molecules22010044

**Published:** 2016-12-29

**Authors:** Min Li, Yu-Ting Cao, Si-Rui Ye, Muhammad Irshad, Teng-Fei Pan, Dong-Liang Qiu

**Affiliations:** College of Horticulture, Fujian Agriculture and Forestry University, Fuzhou 350002, China; liminzyl@sina.com (M.L.); caoyt87@163.com (Y.-T.C.); yesirui@163.com (S.-R.Y.); irshadaup@gmail.com (M.I.); tfpan@fafu.edu.cn (T.-F.P.)

**Keywords:** *Brunfelsia acuminata*, flower color, anthocyanin biosynthesis, chalcone synthase (CHS), RT-qPCR

## Abstract

Chalcone synthase gene (*BaCHS*) from *Brunfelsia acuminata* flowers was isolated using RT-PCR and RACE. The coding region of the gene is 1425-bp with an open reading frame of 1170-bp, 73-bp 5′UTR, and 172-bp 3′UTR. Its deduced protein does not have a signal peptide but does contain a cond_enzyme superfamily domain, and consists of 389 amino acids with a predicted molecular mass of 42,699 Da and a pI of 6.57. The deduced amino acid sequence of BaCHS shares 90%, 88%, 85%, 84% and 79% identity with CHS from *Petunia hybrida*, *Nicotiana tabacum*, *Solanum lycopersicum*, *Capsicum annuum* and *Camellia sinensis*, respectively. The striking color change from dark purple to light purple and ultimately lead to pure white resulted from a decline in anthocyanin content of the petals and was preceded by a decrease in the expression of *BaCHS*. Its gene expression was positively correlated with the contents of anthocyanin (*p* ≤ 0.01).

## 1. Introduction

Flower color is one of the most important characteristics of floriculture plants. Flower color results from the accumulation of flavonoids, carotenoids or betalains, and the type of accumulated pigments largely relies on plant species. Chalcone synthase plays a significant role in flavonoid biosynthesis by catalyzing the stepwise condensation of 4-coumaroylcoenzyme A (CoA) produced from the phenylpropanoid pathway with three acetate units from malonyl-CoA and cyclizing the resulting tetraketide intermediate to naringenin chalcone, which is a flavonoid skeleton [1]. Since the first *CHS* gene was isolated from parsley [2,3], hundreds of *CHS* genes have been cloned from different plants such as *Petunia hybrida* [4], maize [5], *Arabidopsis* [6], *Antirrhinum majus* [7], *Gerbera hybrid* [8], *Vitis vinifera* [9] and *Tulipa fosteriana* [10]. *CHS* genes in *Petunia hybrida* compose a multigene family with twelve *CHS* genes, but only *CHSA* and *CHSJ* are expressed in flower corolla, tube and anthers [11,12]. The expression of *CHS* gene could alter flower pigmentation by transferring antisense chalcone synthase cDNA to *Gerbera hybrid* [13].

Anthocyanins are a type of flavonoid compound responsible for the pink, orange, red, scarlet, purple, blue and cyanic flower colorationx in many plant species. They are widely distributed in higher plants and their structure and biosynthesis have been well studied [14,15].

In addition to cloning and sequence analysis of the chalcone isomerase gene (*CHI*) from *Brunfelsia acuminata* flowers [16], anthocyanin degradation in plants was studied in flowers of *Brunfelsia calicina* (Solanaceae) [17,18]. These flowers change color from dark purple to pure white within the first 2 days of opening. The process is dependent on anthocyanin degradation and de novo synthesis of mRNAs and proteins of peroxidase (BcPrx01) [17,18]. However, to our knowledge, little is known about the *CHS* gene from *Brunfelsia acuminata* flowers and its role in anthocyanin biosynthesis. Therefore, the aim of this study was to isolate the full-length cDNA of the *CHS* from *Brunfelsia acuminata* flowers and analyze the sequences and expression of the cDNA. A better understanding of the mechanism of transcriptional regulation of the *Brunfelsia acuminata CHS* gene may advance genetic engineering of the flower color.

## 2. Results

**Figure 1:**
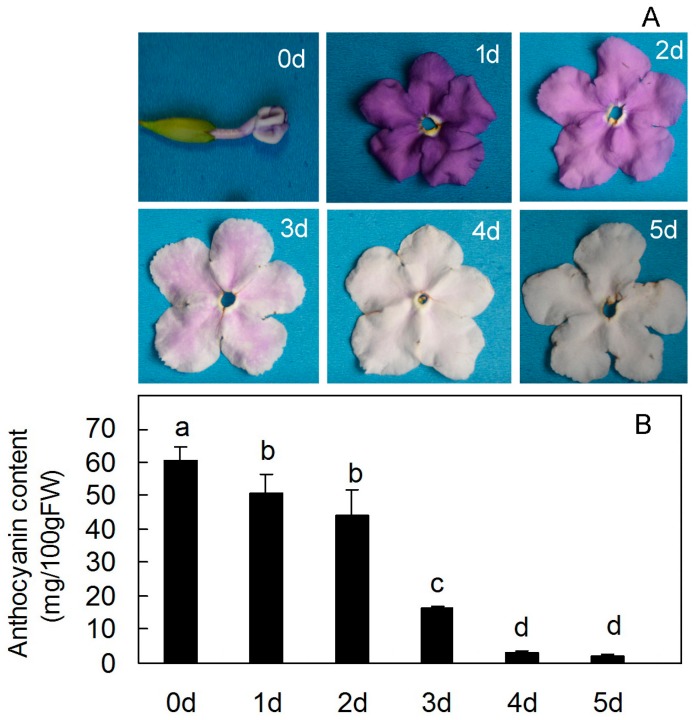
Change in anthocyanin content in petals at sequential stages of flower opening and senescence over 6 days. (**A**) *Brunfelsia acuminata* petals at sequential stages of flower opening and senescence over 6 days; (**B**) The anthocyanin content at sequential stages of flower opening and senescence over 6 days. The data represent means ± SD of three replicate (*n* = 3). Where no error bars are present, The SD was smaller than the size of the symbol. Different letters indicate significant differences (*p* < 0.05).

A particularly interesting feature of *Brunfelsia acuminata* petals is their striking color change from dark purple to light purple and ultimately to pure white as petal coloration develops. These colors changes are associated with a decline in anthocyanin content of the petals. Anthocyanin accumulated to a maximum level at Day 0 and degraded to a minimum level at day 5 (Figure 1).

**Figure 2:**
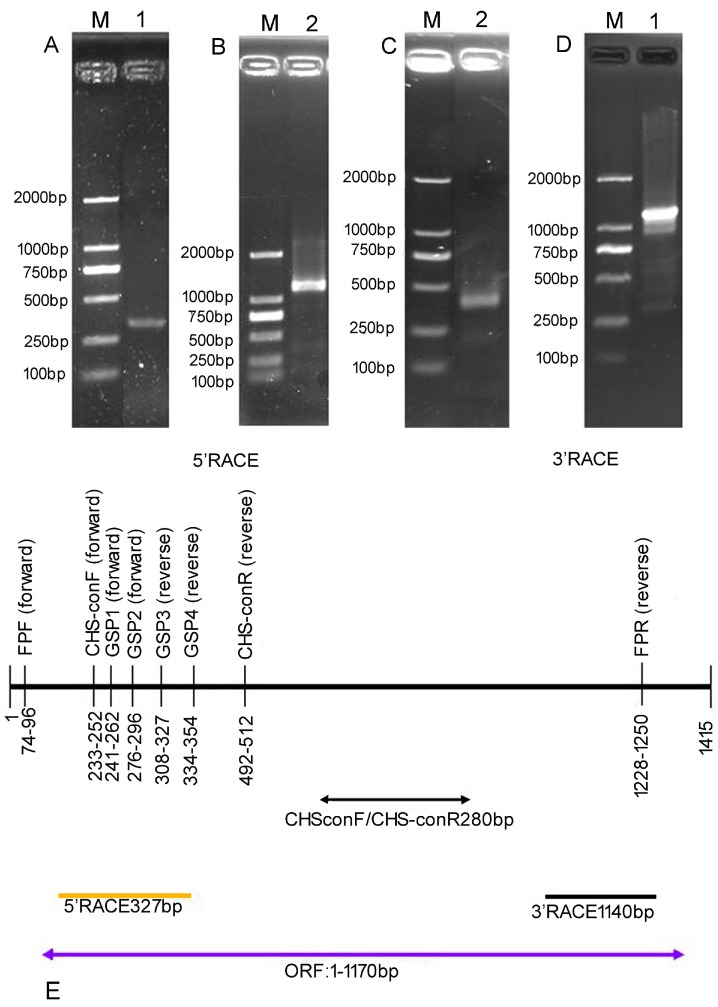
Isolation and identification of *BaCHS*. (**A**) RT-PCR products from the petals of *Brunfelsia acuminata* with primers *CHS*-ConF /*CHS*-ConR for lane 1. Marker: DL-2000 marker; (**B**) 3′RACE-PCR products from the petals of *Brunfelsia acuminata* with GSP1/ GSP2 3′RACE-PCR inner primer; (**C**) 5′RACE-PCR products from the petals of *Brunfelsia acuminata* with GSP3/GSP4 5′RACE-PCR inner primer; (**D**) The full cDNA products of *Brunfelsia acuminata* petals with FPF/FPR primers; (**E**) RT-PCR and RACE-PCR product lengths with different primers.

To identify the major regulatory genes affecting anthocyanin accumulation in *Brunfelsia acuminata* petals, the expression patterns of anthocyanin biosynthetic genes were studied using RT-PCR. The full-length sequence of the *CHS* gene sequence was isolated using a combination of RT-PCR and RACE-PCR. The result demonstrated that the full-length cDNA was assembled as a 1415-bp sequence with a poly (A) tail, and contained an 1170-bp ORF encoding a 389 amino acid protein (Figure 2).

**Figure 3:**
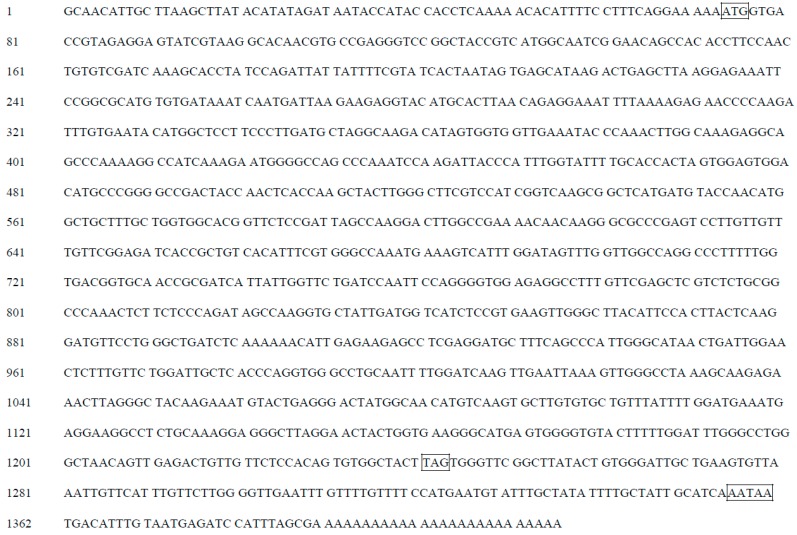
Full length of the sequence of *CHS* cDNA. ATG is start codon; TAG is stop codon; AATAA is the polyadenylation signal. The sequence contain 73 bp 5′UTR and 173 bp 3′UTR, with a 1170 bp open reading frame. The base the third left start codon, and the one following start codon is A and G respectively, which is obey the Kozak sequence.

**Figure 4:**
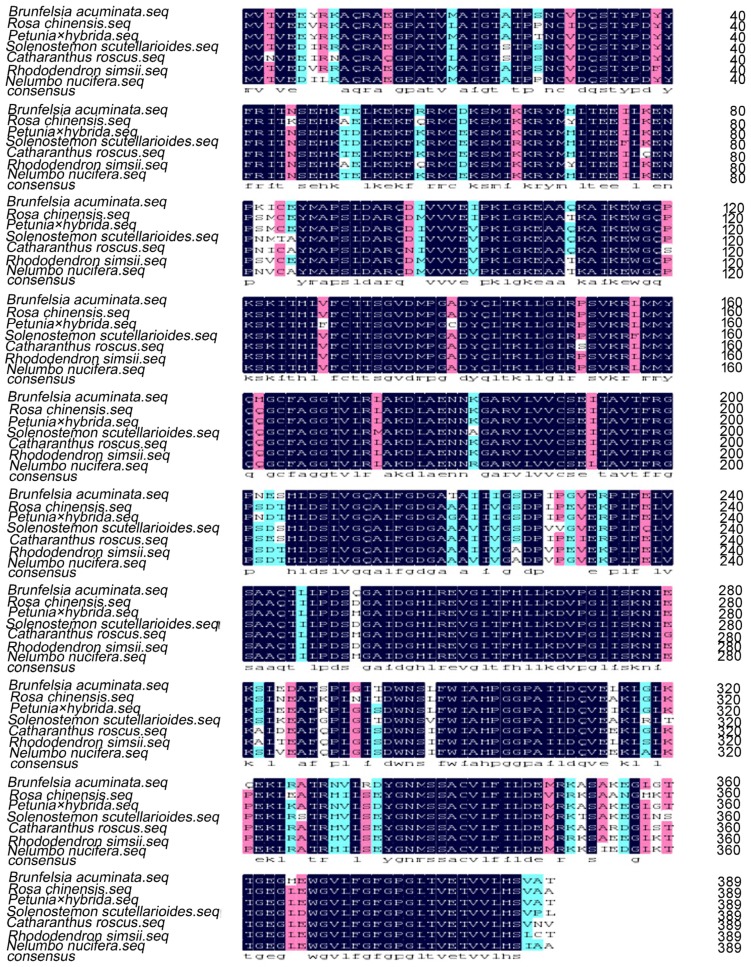
Multiple alignment of predicted amino acid sequences of CHS from different plants. *Petunia hybrida*, CAA32731.1; *Rosa chinensis*, AEC13058.1; *Solenostemon scutellarioides*, ABP57071.1; *Catharanthus roseus*, CAA10511.1; *Rhododendron simsii*, CAC88858.1; *Nelumbo nucifera*, ADD74168.1.

**Figure 5:**
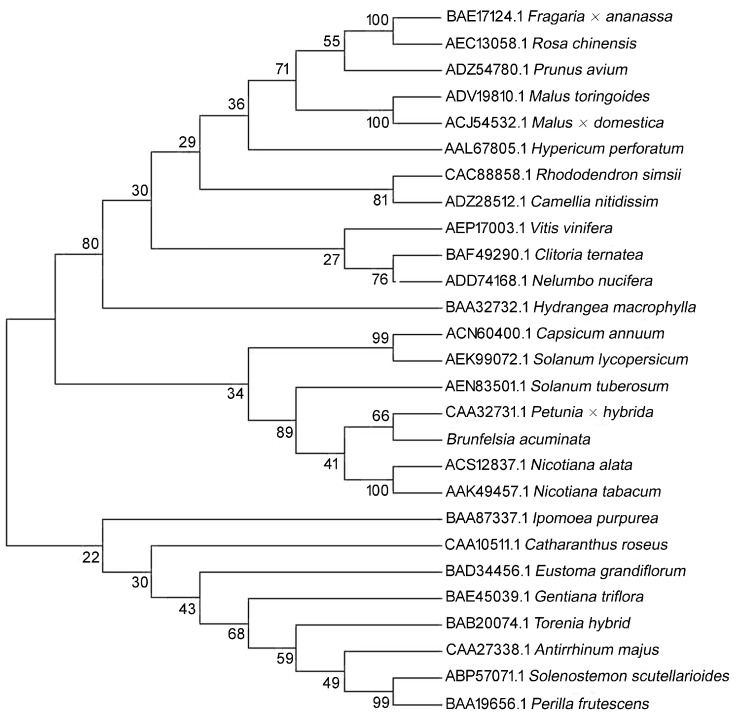
A Phylogenetic tree of deduced amino acid sequences of CHS constructed by software ClustalX 1.8 and MEGA 5.0 from *Brunfelsia acuminata* and from various other plant species. The phylogenetic tree is a neighbor-joining tree calculated by Poisson model, and the bootstrap values are from 1000 replications. The plants’ names and their Genbank accession numbers are all shown in the figure.

**Figure 6:**
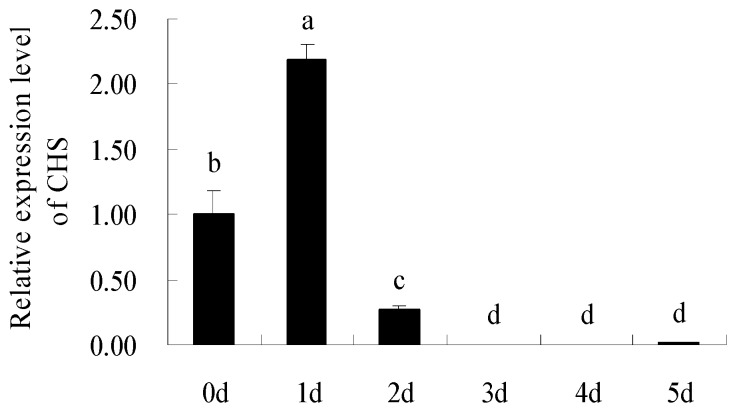
Expression patterns of *BaCHS* transcripts in the petals at sequential stages of flower opening over 6 days of *Brunfelsia acuminata*. Abundances were determined by comparison with an internal reference (*BaActin*) and are shown relative to the expression level on Day 0, which was given a value of 1. The data represent means ± SD of three replicate (*n* = 3). Where no error bars are present, The SD was smaller than the size of the symbol. Different letters indicate significant differences (*p* < 0.05).

There was a 5′ untranslated region of 73-bp upstream from the start codon, and the coding region was followed by a 3′ untranslated region that was 173-bp downstream of the stop codon (Figure 3). Homology search revealed that the cDNA shared 90% identities with *CHS* of *Petunia hybrida* (AF233638.1), 88% identity to *Nicotiana tabacum* (AF311783.1), 85% identities to *Solanum lycopersicum* (NM001247104.1), 84% identity to *Capsicum annuum* (FJ705842.1), and 79% identity to *Camellia sinensis* (AY169403.1). Therefore, this cDNA sequence was designated *BaCHS* with accession serial number JN966986 registered in GenBank. Alignment of protein sequences is shown in Figure 4. BaCHS shared higher sequence identity with the CHS proteins of *Petunia hybrida* (CAA32731.1), *Rosa chinensis* (AEC13058.1), *Solenostemon scutellarioides* (ABP57071.1), *Catharanthus roseus* (CAA10511.1), *Rhododendron simsii* (CAC88858.1), and *Nelumbo nucifera* (ADD74168.1). A high similarity among CHS proteins was observed from residues 40 to 389. The deduced amino acid sequence of BaCHS was phylogenetically analyzed with CHSs from other plants. The phylogenetic tree grouped into several branches (Figure 5). BaCHS was most closely related to CHS from *Petunia hybrida* with higher bootstrap support and grouped into a small branch with CHS from *Petunia hybrida*. As expected, *Brunfelsia acuminata* was closely related to Solanaceae with high bootstrap. *Brunfelsia acuminata*, *Petunia hybrida*, *Nicotiana tabacum*, *Solanum lycopersicum*, *Capsicum annuum*, *Nicotiana tabacum*, *Nicotiana alata*, and *Solanum tuberosum* belong to the family Solanaceae and form one branch according to their higher identity of amino acid sequence throughout the entire coding region (Figure 4). Expression analysis of anthocyanin biosynthetic genes is shown in Figure 6. The transcript levels of *BaCHS* were higher during the bud (Day 0) and initial flowering (Day 1) stages than at later stages (Days 2–5). Anthocyannin accumulation was significantly correlated with the expression level of *CHS* gene. The expression of the genes appeared to suppress by Day 5.

Abundances were determined by comparison with an internal reference (*BaActin*). The data for raw measurement of *BaActin* mRNA expression is shown in Supplementary Materials. The anthocyanin accumulation in the new stem is minimum as compared to leave and flower (Figure 6). Total anthocyanin content was expressed from a weak to strong order thus: flowers > leaves > stems.

## 3. Discussion

In this report, sequence analysis and comparison with *BaCHS* revealed that the ORF was 1170 bp in length and putatively encoded a polypeptide of 389 amino acids, which had high similarities (84%–90%) with CHSs from other plant species by blasting in NCBI. A putative amino acid sequence of BaCHS contained active amino-acid residues that are highly conserved among all CHS sequences, and *BaCHS* is a homolog of the *CHS* gene, with its protein being a typical CHS protein. The color changes of the *Brunfelsia acuminata* flower could be explained by several reasons. These may include the spatiotemporal regulation of anthocyanin biosynthetic genes and/or their regulatory genes, or mutational effects on the structural genes encoding enzymes for anthocyanin biosynthesis. Simultaneously, the expression levels of *CHS* and the external factors are closely related, such as light, temperature, water and so on. For example, light is the key environmental factor, without light, it will hinder the synthesis of anthocyanins in flower petals and inhibit the expression of *CHS* gene, eventually leading to corolla coloring [19]. The stronger the light, the more anthocyanins accumulate. Blue light and ultraviolet light are the most effective light qualities for the synthesis of anthocyanin [20]. Temperature is too high or too low, the stability of anthocyanins will be affected [21]. However, we focused on the first possibility because purple petal pigmentation decreased dramatically with the petal development (Figure 1). To determine the expression patterns of three anthocyanin biosynthetic genes in petal in detail, quantitative RT-PCR was performed using total RNA isolated from bud and petals. CHS controls the first step in anthocyanin biosynthesis. Thus, we assume that its expression is high in the petal of the *Brunfelsia acuminata* flower. It has been documented that CHS is correlated with anthocyanin synthesis in *Malus* [22], *Morus alba* [14], *Vitis vinifera* [23,24], *Pyrus* [25], and *Fragaria ananassa* [26,27,28].

As expected, *CHS* transcript levels increased to a maximum in Day 1 flowers that were beginning to change color and thereafter decreased as petal coloration continued to develop. The abundance of *CHS* mRNA increased more than 2-fold between Days 0 and 1 before decreasing to the lowest level by Day 5 (Figure 6). RT-qPCR analysis demonstrated that the pattern of *CHS* gene expression in *Brunfelsia acuminata* was positively correlated with the contents of anthocyanin (*p* ≤ 0.01). Therefore, the results suggested that the color of the petal was attributed to a high expression of *CHS*. The expression decreased significantly from Day 3 to Day 5, and the anthocyanin content dropped sharply. Thus, the petal color disappeared on the 5th day of flowering. Anthocyanin degradation and lower level expression of *CHS* gene for the enzyme involved in anthocyanin synthesis appeared to end by Day 5, indicating that the bud and initial flowering stages (Day 0–1) represent the key phase of anthocyanin biosynthesis and that the degradation of anthocyanin started on Day 2. Our results also indicated that *BaCHS* expression and total anthocyanin accumulation in different tissues clearly showed the different patterns. Modulation of the *Brunfelsia acuminata CHS* gene may advance genetic engineering of flower color.

In addition, it’s worth noting that the chalcone isomerase (CHI) catalyzing the next step was previously identified in *B. acuminate* [16]. Both CHS and CHI are key enzymes in anthocyanin biosynthesis. CHS catalyzes the first step of the reaction, and the CHI catalyzes the second reaction. Their changes in expression of level or loss of function and inactivation of the enzyme will directly affect the levels of flavonol compounds [29]. The protein accumulation of CHS and CHI are coordinately. This effect is due to the coordinative expression of *CHI* and *CHS* mRNA [30]. The comparison of the expression of these two genes during flower maturation will be done in the future.

## 4. Materials and Methods

The *Brunfelsia acuminata* was grown in the campus of Fujian Agriculture and Forestry University. Flower buds opened with dark purple petals that changed to purple and then to light purple and then to white as the flower aged during a 6-day lifespan. Flower developmental stages were divided into bud stage as Day 0 and flower open as Day 1 and the flower color fading away as Day 5. Flowers were tagged based on stages. The tagged flowers at different developmental stages were then harvested daily for experimental evaluation of petal coloration and gene expression. All samples were immediately frozen in liquid nitrogen and stored at −80 °C until use. Primers used in the study of *BaCHS* are shown in Table 1.

Total anthocyanin contents were determined according to the method of Weiss and Halevy [31] with slight modifications. In brief, 0.2 g of each tissue was ground in liquid nitrogen and total anthocyanins were extracted with HCl/ethanol (1:99, *v*/*v*) at 60 °C for 4 h. The samples were centrifuged at 12,000 rpm for 20 min. The supernatants were retained and the absorbance values were determined using UV spectrophotometry at 535 nm and 650 nm. The anthocyanin content was measured in milligrams per gram fresh weight tissue [32].

Genomic DNA was extracted from the leaves of *Brunfelsia acuminata* using DNA extraction kits and following the manufacturer′s instructions (Biospin, Beijing, China), and total RNA was extracted separately from petals at different developmental stages by using RNA extraction kits (BioTeke, Beijing, China) [16]. The quality and concentration of the RNA and DNA were determined by agarose gel electrophoresis and spectrophotometer analysis. To amplify the conserved fragments of *BaCHS*, degenerate oligonucleotide primers were designed based on conserved amino acid regions of other plant species obtained from the GenBank database using NCBI BLAST. RT-PCR was performed using the RevertAidTM First-Strand cDNA Synthesis Kit (Fermentas, Shanghai, China). The PCR product was purified and cloned into the pMD18-T vector (Takara, Dalian, China), and then sequenced at Biosun Company (Shanghai, China). Subsequent BLAST results confirmed that the amplified product was a partial fragment of the BaCHS gene. The 3′ end and 5′ end sequences were amplified using Super SMARTerTM RACE cDNA Amplification Kits (Clontech, Mountain View, CA, USA), and the open reading frame (ORF) was amplified by RT-PCR. The primers for 3′-RACE and 5′-RACE were designed to the originally conserved region, and the ORF primers were designed by cDNA assembly with the conserved region, 3′ end sequence and 5′ end sequence. PCR amplified products were examined on a 1.0% agarose gel by electrophoresis. Then, the products were cloned into the pMD-18T vector (Takara, Dalian, China) and sent for sequencing at the Biosun Company. After comparing and aligning the sequences of the 5′-RACE, 3′-RACE, and the internal region products, the full-length cDNA sequence of *BaCHS* was obtained through RT-PCR. The PCR product was purified and cloned into the pMD18-T vector followed by sequencing. Subsequently, the full-length cDNA of *BaCHS* was analyzed for molecular characterization.

For quantitative reverse-transcription PCR (RT-qPCR), the cDNA was synthesized using the PrimeScript^®^ RT Reagent Kit according to the manufacturer′s instructions (Takara, Dalian, China), and transcripts were amplified with an SYBR Premix ExTaqTM II kit (Takara, Dalian, China). The relative gene expression was calculated using the 2^−^^△△Ct^ method [33]. RT-qPCR experiments were performed in triplicate. The *BaActin* gene from *Brunfelsia acuminata* was used as an internal reference as shown in Table 1.

The data determined in triplicate were analyzed by analysis of variance (ANOVA) using SPSS version 16.0 (IBM, Armonk, NY, USA)). The significance of differences was determined according to Duncan′s multiple range test (DMRT). *p* values ≤ 0.05 are considered to be significant. The results are presented as the means ± SD. Pearson correlation was calculated by SPSS version 16.0 with 2-tailed test of significance.

## Figures and Tables

**Table 1:** Primers used in the study of BaCHS and RT-qPCR.

Primer	Primer Sequence	Use
*CHS*-conF(forward)	5′-GAGAARTTCMRGCGCATGTGYG-3′	cDNA
*CHS*-conR(reverse)	5′-GCTTGGTGAGYTGRTAGTCRG-3′	
GSP1(forward)	5′-CGGCGCATGTGTGATAAATCA-3′	3′RACE-PCR
GSP2(forward)	5′-GGTACATGCACTTAACAGAGG-3′	
UPM	5′-CTAATACGACTCACTATAGGGCAAGCAGTGGTATCAACGCAGAGT-3′	
GSP3(reverse)	5′-TCACAAATCTTGGGGTTCTC-3′	5′RACE-PCR
GSP4(reverse)	5′-CTAGCATCAAGGGAAGGAGCC-3′	
UPM	5′-CTAATACGACTCACTATAGGGCAAGCAGTGGTATCAACGCAGAGT-3′	
FPF(forward)	5′-ATGGTGACCGTAGAGGAGTATCG-3′	Full-length
FPR (reverse)	5′-GAACCCACTAAGTAGCCACACTG-3′	
*CHS*-F(forward)	5′-CAAAGGAGGGCTTAGGAACTACT-3′	RT-qPCR
*CHS*-R(reverse)	5′-CAAATTCAACCCCAAGAACAAATGA-3′	
*BaActin*-F(forward)	5′-AACCATAAACGATNCCGACCAG-3′	
*BaActin*-R(reverse)	5′-NCTTGCGACCATACTCCC-3′	

## Supplementary Materials

Supplementary materials can be accessed at: http://www.mdpi.com/1420-3049/22/1/44/s1.
