# Supplementary material for: Isolation of CHS Gene from Brunfelsia acuminata Flowers and Its Regulation in Anthocyanin Biosysthesis
Source: Molecules. 2016 Dec 29;22(1):44. doi: 10.3390/molecules22010044 (PMC6155851; doi:10.3390/molecules22010044)
Supplement: Supplementary file 1 [file molecules-22-00044-s001.pdf]

Supplementary Materials Isolation of *CHS* Gene from *Brunfelsia acuminata* Flowers and Its Regulation in Anthocyanin Biosysthesis

Min Li, Yu-Ting Cao, Si-Rui Ye, Muhammad Irshad, Teng-Fei Pan, and Dong-Liang Qiu

Table S1. The data for raw measurement of *BaActin* mRNA expression.

Fluor	Target	Content	Sample	Cq
SYBR	*BaActin*	Unkn	0d	10.28
SYBR	*BaActin*	Unkn	0d	10.09
SYBR	*BaActin*	Unkn	0d	10.51
SYBR	*BaActin*	Unkn	1d	10.22
SYBR	*BaActin*	Unkn	1d	10.11
SYBR	*BaActin*	Unkn	1d	10.01
SYBR	*BaActin*	Unkn	2d	11.08
SYBR	*BaActin*	Unkn	2d	11.24
SYBR	*BaActin*	Unkn	2d	11.34
SYBR	*BaActin*	Unkn	3d	11.24
SYBR	*BaActin*	Unkn	3d	11.03
SYBR	*BaActin*	Unkn	3d	11.23
SYBR	*BaActin*	Unkn	4d	10.78
SYBR	*BaActin*	Unkn	4d	11.15
SYBR	*BaActin*	Unkn	4d	10.75
SYBR	*BaActin*	Unkn	5d	11.19
SYBR	*BaActin*	Unkn	5d	11.44
SYBR	*BaActin*	Unkn	5d	11.28
